# The Adulteration of Drugs

**Published:** 1895-09

**Authors:** Willis G. Tucker


THE
AMERICAN JOURNAL
—OF—
DENTAL SCIENCE.

VOL. XXIX. BALTIMORE, SEPTEMBER, 1895. No. 5.

ARTICLE I.

### THE ADULTERATION OF DRUGS.

BY WILLIS G. TUCKER, M. D., PH. D.

A Lecture delivered before the Brooklyn Institute, May 24, '95.

Pure air to breathe, and pure food and water to eat and drink, are perhaps in a sense matters of more importance to the community than pure drugs to use in sickness, because the normal condition of the body is health, and if this be maintained by respecting the laws of nature, and living well-ordered lives amid sanitary surroundings, we shall have little need of medicine, but, unfortunately, many of us do not respect these laws, or we are unwilling or unable to live in a healthful environment. Through hereditary influences, moreover, for which we are in no wise responsible, many of us come into the world badly handicapped in the matter of health, and, hence, as a result of unwise living, bad surroundings or natural taint, we fall a prey to disease and need the physician's aid. Hold what view we may as to the real efficacy of the medicines which he administers, the great majority of us take more or less of them in the course of our lives, and when we *do* take them we are naturally desirous that they be pure and properly compounded. Absolute purity or precise strength may not in all cases be matters of supreme importance, but the feeling is not only natural, but entirely justifiable, that, if medicine is in any sense a science, the medicinal agents employed in the treatment of disease should be administered with some approach to scientific precision. Looked at in this light, therefore, nothing is unimportant, and the pharmacist who would be the true ally of the ideal physician must take this view of his calling and practice it as a profession and not as a trade. In other words, he, no less than the physician, must be imbued with the scientific spirit, and regard accuracy and precision in the practice of his art as of no less importance than business integrity in the conduct of his affairs.

The first question that we may well ask is: What constitutes adulteration? Our latest dictionary, the "Standard" says that to adulterate is to "make impure by the admixture of other, or baser, ingredients," and this definition is practically the same as that given by most other lexicographers, and it is the meaning which we ordinarily attach to the term. But the word as used in our statutes, and by analysts, has a much broader, and often a very different significance. A great many articles *are* intentionally debased by the addition of cheap make-weights with fraudulent intent. Thus, among foods, for example, glucose is added to maple syrup; starch and gypsum to cheap confectionery; roasted cereals to ground coffee, and all sorts of rubbish to ground spices, but of this kind of adulteration, there is, according to my experience, comparatively little in the manufacture and preparation of drugs. The adulteration of drugs does not generally consist in the direct and intentional addition of foreign material, but in the substitution of articles of inferior quality, or of inferior and sometimes of executive strength. The "Public Health Law" of this state, being chapter 661 of the Laws of 1893, defines in section 41, article 111, adulteration, in the case of drugs, as follows: "An article shall be deemed to be adulterated within the meaning of this act: 1. If when sold under or by a name recognized in the United States Pharmacopœia, it differs from the standard of strength, quality or purity laid down therein. 2. If when sold under or by a name not recognized by the United States Pharmacopœia, but which is found in some other pharmacopœia or standard work on materia medica, it differs materially from the standarn of strength, quality or purity laid down in such work. 3. If its strength or purity fall below the professed standard under which it is sold." Therefore if one article be substituted for another, as common safflower for crocus; or if an article, originally of good quality, has deteriorated by age or exposure, as may happen in the case of such articles as diluted hydrocyanic acid, ammonium carbonate, or magnesia; or if, owing to careless or improper manufacture, an article contains foreign matter, whether intentionally added or not, as alkaline carbonates in the bromides or iodides, or calcium sulphate in precipitated sulphur; or if it be deficient in strength, or of excessive strength, as is often the case with the diluted acids—then such an article is adulterated within the meaning of the act. It is well that this be distinctly understood, because the common opinion is that only those articles are adulterated which are intentionally debased, and that such adulteration is very common, whereas the fact is that such an adulteration of drugs is by no means extensively practiced. I do not say that it does not occur, but it is by no means as common as is generally supposed. The inferior drugs that are sold in our stores are for the most part inferior because articles of lower grade, perhaps not even included in the pharmacopœia, have been purchased because cheaper, or for the reason that they have been carelessly or ignorantly prepared or preserved, and not because of intentional admixture of foreign material, and it has taken pharmacists a long time to understand this matter and recognize the fact that they are responsible for the goods they dispense, and that it is their bounden duty to know the quality of the articles in which they deal. They cannot shift this responsibility to the manufacturer or the wholesaler. It is the business of the pharmacist to know the different grades of the articles which constitute his stock in trade; to select them with careful discrimination; to preserve them in a proper manner; to test their quality, if need be, and adjust their strength, when necessary, and to use due care in the preparation of such articles as he himself manufactures, so that they shall be of the quality and strength that the pharmacopœia demands or established custom requires. In other words, the pharmacist should be a skilled workman, and not a mere retailer of commodities. The public regard him as proficient in his particular calling and have a right to expect expert service when they present prescriptions for his compounding, and if he is satisfied to dispense whatever he buys from the maker or the wholesaler, without inquiry or warrant of any kind, or test of his own making, he has no right to claim the title of pharmacist, nor to charge for the materials he furnishes more than they are worth in bulk with a fair retailer's profit added thereto. The public are ready to pay and expect to pay him not only for the drugs he compounds, but for the skill employed in selecting, preparing, preserving and properly compounding them, and if this skill be lacking, they are defrauded and the imposition is the more culpable because the matter may be one in which health or even life is at stake.

This is perhaps not the place to discuss at any length the duties and responsibilities of the pharmacist, or the present condition of American pharmacy, but a few words upon some of these matters may not be out of place in this connection. We have said that the pharmacist should be imbued with the scientific spirit, and from the outset, he should be taught to regard his calling as a profession. It is unfortunate that he is at present, in a sense, required to combine with his legitimate trade in drugs, a trade in sundries, some of which are in no way related to his real business. There is no reason why he should deal in non-medicinal beverages and fancy goods like toilet articles, to say nothing of cigars, books and periodicals, and the odds and ends of all kinds, which, while adding to the volume of his trade, detract from the dignity of his legitimate calling and the proper conduct of his business. But so it is, at present, and all that he can perhaps do is to magnify in every proper way the importance of that part of his work which *is* legitimate, and minimize so far as he can that part of it, the very existence of which is to be regarded as an outgrowth or excrescence. If the necessities of the case require him to deal in such articles as have been named, let him at all events so arrange his store and dispose of his stock that they shall not occupy the place of prominence. There are some drug stores, the true nature of which it would be difficult to determine on a hasty inspection, were it not for the decorated jars and colored show-bottles which adorn the windows, or the gilded and illuminated mortar over the entrance, which serves as a sign by day and a beacon by night.

The outlook in some respects is encouraging, but influences are at work which we may hope will eventually raise the business of the druggist to the plane upon which it ought, from every right consideration, to be established, and among these may be mentioned the legislative action which has been taken, within recent years, in our own and in other states. We can not hope that legislation will accomplish any great reforms suddenly. Too much should not be expected from such action, as we shall presently more particularly point out, but the fact is by no means to be ignored that in forty-four of the states and territories we now have pharmacy laws, which, to some extent at least, regulates the sale of drugs and poisons, and prohibit adulteration, and these laws, though enforced with varying degrees of efficiency, have yet done something, and may be expected in the course of time to do more, to elevate American pharmacy and protect the public. The examination, registration and licensing of pharmacists, to the end that only those who have shown themselves capable shall be allowed to dispense medicines, is a step in the right direction, as are also definitions of adulteration and the legal establishment of standards.

Most of these pharmacy laws follow pretty closely the English statutes, and though in some cases their enactment has been opposed on the ground that they interfered with individual rights and unnecessarily hampered business, they are now pretty generally conceded to be right in principle, and on the whole, productive of fairly good results in practice. Professor Oldberg of Chicago has recently offered for consideration and discussion a draft of a proposed pharmacy law in which provision is made for the licensing of stores of different grades, as follows: 1. Those under the management of a registered pharmacist in which poisons and potent remedies may be sold, to be known as pharmacies of the first class: 2. Those under the charge of a registered pharmacist, or assistant pharmacist, in which poisons and potent remedies shall not be permitted to be sold, to be known as pharmacies of the second class; 3. Those conducted by licensed druggists (as defined in the act) in which the preparation and compounding of medicines is prohibited, to be known as licensed drug-stores. The principle involved in this proposed legislation seems to me to be correct, but whether such a classification could be practically made and maintained I do not pretend to say. Be this as it may, the State is certainly coming to deal more definitely than ever before with the relations existing between its citizens, and many new laws regulating the practice of the professions and the conduct of many of the trades, have, during recent years, been enacted. Our pharmacy laws belong to this class, and it is all-important that pharmacists should understand the nature of the evolution which is taking place, and should conform to the new demands which are made upon them. Our colleges of pharmacy have no unimportant part to play in bringing about the reforms which are so greatly to be desired. They should not only impart scientific instruction, but they should explain the legal provisions which govern the practice of pharmacy and impress upon their students the fact that they occupy an important place in the community, and are, in a sense, public servants with well-defined and highly important duties to perform. In a state like ours, in which the duties and responsibilities of the pharmacist are, to a certain extent at least, defined, he can not plead ignorance of the law as an excuse for non-compliance with its requirements. With new responsibilities come new opportunities and new honors also, and he is lacking in proper self-respect and proper regard for the dignity of his calling if he seeks to evade these responsibilities and prefers to fill the place of a tradesman rather than stand forth as a skilled workman in a scientific pursuit. And as the demands upon him are multiplied, so should his compensation be increased. His charges for services rendered should be proportioned to the skill required of him, and to the investment of time and of money which he has made in preparing himself to serve the public properly and legitimately. May we not hope that the time is not far distant when "cut rate" drug stores will be no more; unfair competition a thing of the past; charlatanism of all kinds banished from this business, and the practice of pharmacy, conducted by educated men alone, raised to the place which it ought to occupy among scientific pursuits?

That many abuses exist at present is implied in what has been said, but for many of these, the pharmacist himself is by no means responsible. Manufacturers have flooded the market with proprietary preparations and quack nostrums of all kinds, and these things, many of them of no value, the pharmacist is expected to keep in stock. In this respect, he occupies a very uncomfortable position, for if he denounces them as frauds and refuses to keep them, trade goes elsewhere and seeks those who are less punctilious and less deserving, and if he continues to sell them he feels that he degrades his calling. Another class of manufacturers, the manufacturing chemists, have put upon the market a bewildering variety of pharmaceutical preparations, the sale of which is, in many cases, supposed to be made only upon the physician's prescription. These are frequently changed, and they are multiplied from day to day. The manufacturer sends his agent, who calls upon the physician in person, recommending his products and leaving samples for his use, and by this personal solicitation, combined with the generous distribution of samples and lavish advertising in the journals, his goods are pushed into prominence, and the pharmacist must carry them in his stock, often at great risk, and not infrequently at a loss, or lose his patronage. I shall not attempt to say how these abuses may be remedied, merely suggesting that as a result of concerted action among druggists, which would be possible, if, as a class, they were better organized for the protection of their own interests, and with better legislation, perhaps, the sale of quack nostrums and cure-alls, at the least, might be greatly lessened if not largely suppressed. The other evil referred to, the multiplication of non-official pharmaceutical preparations, is one not so easy to deal with. Many of them are of great value and constitute welcome additions to our materia medica, but their name is legion, and no one, I take it, will affirm that we need them all. So rapidly, however, is their use increasing, that many physicians depend very largely upon them, and seldom write prescriptions in the true sense, in which recognized medicinal agents are, either singly or combined with each other, prescribed with special regard to the particular case under treatment. Instead of so doing, they use some laxative or alterative; some tonic or sedative; some febrifuge or stimulant, of the real nature and constituents of which they have often but a general and very hazy knowledge, kindly obliging the manufacturer by specifying so and so's make in the prescription. Now I maintain that this is very largely wrong, and that such a method of prescribing leads to many abuses and degrades medicine. It leads to the unauthorized duplication of prescriptions and to the general use, and often gross abuse, of remedies which ought to be taken only upon a physician's advice, and it limits the need for trained pharmacists and takes from such, a large share of their legitimate business. The sale of tablets, which has grown to such proportions of late, illustrates my meaning. Reputable physicians tell me that they find themselves, almost against their will, prescribing combinations of remedies in this form in a manner almost as arbitrary as if they ordered the use of somebody's purgative pellets, anti-neuralgic pills or sedative elixir. They are so convenient to dispense or prescribe that they are led to abandon the use of standard preparations, and in their stead, dole out these handsomely prepared and often highly ornamental discs, the composition of which they often forget and perhaps never have known. And their patients know them by name, get them in quantity, use them *ad libitum* and recommend them to their friends as occasion seems to require.

For abuses of this kind, physicians are themselves to blame and not the pharmacists, and it seems to me high time that this matter received the attention which its importance demands. If medicine is a science, and pharmacy, at the least an art, is it not time that the physician returned to a more rational method in the treatment of his cases? If not, he might as well abandon the study of materia medica and pharmacology; fill his case with empirical remedies, and admit that he has little use for the service of the educated pharmacist. But I trust that he will be reluctant so to do, and I look for an agitation of this matter which will lead to an abandonment of many of the preparations he now employs and a return to a more rational method of treatment. This is a matter which physicians and pharmacists may well consider together.

The New York State Board of Health was created by legislative enactment in 1880, and prior to that time there had been little legislation in this state on the subject of food adulteration, and practically none at all relating to drugs. In 1881 "an act to prevent the adulteration of food and drugs" was passed, being chapter 407, and it was made the duty of the State Board of Health "to take cognizance of the interests of the public as it relates to the sale of food and drugs and the adulterations of the same, and make all necessary investigations and inquiries relating thereto." Under this statute the Board appointed ten analysts to whom were entrusted investigations along different lines and voluminous reports were made by these analysts and printed in the second annual report of the Board. At a later date the number of analysts was reduced, and in 1891 the State Board of Health Laboratory was established and the entire chemical work of the Board was placed in the hands of the present director. Having been connected with this work from the outset, I have had occasion to examine a very large number of samples of drugs, more than 10,000 in all, and as these samples have been collected in almost all parts of the state and represent a wide variety of articles, it is possible that some of the results obtained may be of interest if briefly stated.

The term "drug" as used in the statute referred to includes "all remedies for external and internal use," and the public health of 1893, which repealed the law of 1881, gives a similar definition. The work which has been done by the State Board, however, has been confined almost exclusively to the examination of pharmacopœial articles, and the more espically as the pharmacopœia is recognized as an authoritative work, both by custom and legal enactment, and that we have in this work a definite standard of strength, quality or purity, for most of the articles included therein.

During the years 1861-1894 inclusive, 8,305 samples of drugs, chiefly pharmacopœial, were examined. Attention will presently be called to some of these, but in a general, way, it may be said at the outset that the object of the work has been not the determination of the proportion of adulterated to pure drugs as found upon the market, but the exposure of actual adulteration, substitution, sophistication and carelessness in the preparation of common drugs and pharmacopœial compounds; the warning of dealers and the publication of the results of the examination of samples obtained from them, and the information of the public. Therefore, no attempt has been made to collect a wide varicty of articles, and such as are known to be seldom adulterated or of inferior quality, have not been collected, but such substances have been selected as would best test the knowledge, accuracy and integrity of the dealer. In the collection of samples, in every instance, the collector has tendered a written order, and this order has given the official names of the articles called for and the amount desired.

Now, the results of the examination of these 8,305 samples will, at first sight, be surprising. Of the total number, but 47.4 per cent. could fairly be rated as of good qnality; 14.8 per cent. were of fair quality; 29.6 per cent. were inferior; 4.4 per cent. were of excessive strength, and 3.8 per cent. consisted of articles wrongly sold, or not as called for. These percentages, however, by no means represent the proportions of good, bad or indifferent drugs on the market and on sale at the stores, since only those articles which were considered likely to be adulterated or known to be frequently ot inferior quality, were collected. Had samples of drugs and pharmaceutical preparations been selected at random, the proportion of pure and good articles would have been very much larger.

Concerning the above classification or rating of the samples examined, it may be said that in the case of those articles in which the pharmacopœia established a definite standard of strength, as for instance in the case of the diluted acids, like hydrocyanic or hydrobromic, then an article must be either good or bad according as it does or does not conform to this standard, but such a method of rating would be manifestly unjust. It is not to be expected that medicinal articles can be prepared with the same precision that a chemist would employ in the making of a standard solution, and we therefore allow for some variation within remarkable limits. In our reports, samples are classed as of "good quality" when they fulfill the requirements of the United States Pharmacopæia or vary from the same only in some unimportant respect; of "fair quality," if, while not fully up to the pharmacopœial standard, they are evidently neither intentionally adulterated nor decidedly below such standard, and of "inferior quality," if clearly adulterated or falsified, lacking in any important constituent, deficient in strength from improper manufacture, partial or complete decomposition or other causes, or containing an undue amount of impurity. In some cases, through ignorance or intent, a wrong article is sold or some inferior article of a nature similar to that called for is substituted, and such samples are classed under the head of "not as called for." Articles like the diluted acids, possessing excessive strength, are classed under that head.—*Albany Medical Annals.*

